# Expression Pattern of Alternative Splicing Variants of Human Telomerase Reverse Transcriptase (hTERT) in Cancer Cell Lines Was not Associated with the Origin of the Cells

**Published:** 2015

**Authors:** Mohsen Khosravi- Maharlooei, Mansooreh Jaberipour, Ahmad Hosseini Tashnizi, Armin Attar, Fatemeh Amirmoezi, Mojtaba Habibagahi

**Affiliations:** 1 *Student Research Committee, Cell and Molecular Medicine Research Group, Shiraz University of Medical Sciences, Shiraz, Iran.*; 2 *Shiraz Institute for Cancer Research, Shiraz University of Medical Sciences, Shiraz, Iran.*; 3 *Immunotherapy Laboratory, Department of Immunology, School of Medicine, Shiraz University of Medical Sciences, Shiraz, Iran.*

**Keywords:** Alternative splicing, cancer cell line, hTERT variants, proliferation capacity, telomerase activity

## Abstract

Telomerase and systems controlling their activity have been of great attention. There are controversies regarding the role of the alternative splicing forms of the human telomerase reverse transcriptase (hTERT), the catalytic subunit of telomerase. Therefore, the correlation between telomerase enzyme activity, the abundance of alternatively spliced variants of hTERT and doubling time of a series of cancer cell lines originated from hematopoietic, breast, colorectal, neural, ovarian, lung, kidney, bladder, prostate and head and neck cancers were investigated. Expression levels of four different variants of hTERT (the full length, α-deletion, β-deletion and α/β-deletion) were quantitatively measured by real time PCR. Telomerase activity was determined by the telomerase repeat amplification protocol (TRAP) while doubling time of the cells measured by plotting growth curves. Results showed high diversity in the relative proportions of hTERT transcripts while the majority of the cells expressed the full length variant as the main transcript. Telomerase activity could not be detected in all cells. Relative assessment of hTERT expression showed greater expression of the α-deleted variant in the telomerase negative cells (P= 0.04). Those cells possessed the α/β-deleted variant to a smaller extent when compared to the cells with telomerase activity. Greater association between full length spliced variant and β-variant expression was observed in cells presenting telomerase activity (P= 0.0007, r= 0.74). High degrees of variation among the studied cells regarding the pattern of hTERT expression were present. In spite that, the regulatory roles of hTERT on telomerase activity is still a potential to be utilized as targets for cancer therapies.

Eukaryotic cells with linear chromosomes face “end replication problem” during cell division as DNA polymerases cannot replicate fully to the very end of one of the DNA strands. In this way, chromosomes lose 50 to 200 base pairs (bp) of their terminal nucleotides (telomere) in every cell division. The accumulation of these chromosomal erosions cause cellular senescence and stop further cellular division. However, eukaryotic cells use an enzyme called telomerase to solve this problem. In this way, during embryonic stages, active telomerase adds up some nonfunctional DNA repeats to the telomeric ends, to keep the critical length of DNA during multiple cell divisions (1). However, as this enzyme is inactive in most adult cells, somatic cells have a limited replica-tive capacity and become senescent after a finite numbers of cell divisions (2). Due to the constant replication of cancer cells, it would be obvious to consider abnormal over-expression of telomerase as an important process of carcinogenesis. In fact, reactivation of telomera-ses has been found in most cancer cells, but not in adjacent normal cells, which makes telomerase as suitable therapeutic anti cancer target. Telomerase is a ribonucleoprotein enzyme which consists of two major components. Human telomerase reverse transcriptase (hTERT) is the protein part and human telomerase RNA (hTR) acts as RNA template. The hTERT gene consists of about 37 kb in genomic DNA from which 33 kb constitute intronic sequence. The remaining 4 kb carries 16 exons to make the hTERT mRNA transcript (3-4). In general, expression of telomerase is a controlled process; however, not all controlling mechanisms have been elucidated. It has been shown that hTR can be expressed in cells regardless of telomerase enzyme activity, while hTERT is only expressed in cells with telomerase activity (5). Several reports have shown strong correlation between telomerase activity and hTERT mRNA expression in different tumor types suggesting that transcription of the hTERT gene can act as a major regulatory step (6). In fact, exogenous over expression of hTERT could immortalize a non neoplastic cell which supported the ideas that hTERT expression can act as a limiting factor for telomerase activity and as a target for cancer therapy as well (7-8). In addition to that, post transcriptional modifications of hTERT have been proposed to alter telomerase activity in cells (9). hTERT transcript is known to have seven alternative splicing sites, from which multiple tissue specific and possibly disease specific alternative transcripts could be produced (10). Therefore, the expression levels of hTERT variants could be the rate-limiting factor in telomerase activity (11). Several reports demonstrated the significant role of alternative splicing variants of hTERT as a factor regulating telomerase activity (12), although controversies exist (9, 13). The most known variants of hTERT mRNA are alpha deletion variant (α-), beta deletion variant (β-) and alpha/beta deletion variant (α/β-) (14). Experimental evidence showed that clones with β-deletion variant could not induce telomerase activity (15). On the other hand, some other evidence showed that different alternatively spliced variants could promote cell proliferation and reduced apoptosis independent of the enzymatic activity of telomerase (16-17).

Therefore, in this study we correlated the expression levels of different telomerase variants and the telomerase activity with cellular doubling time, as a marker of replication capacity, in a series of cancer cell lines. With different levels of expression of each variant, we could show differences between cells with detectable telomerase activity and those with no enzyme activity. Such findings may help to better understand the role of different variants of hTERT and provide information to better utilize telomerase as target in cancer therapy approaches

**Table 1:** List of the cancer cell lines and their origin that were used in the study

**Hematopoietic**		**Brain cancer cell lines**	
**Cell line**	**Origin and Type**	**Cell line**	**Origin and Type**
Jurkat	T cells	U87MG	Glioblastoma-astrocytoma
		A172	Glioblastoma
		**Lung cancer cell lines**	
KG-1	Acute myeloid leukemia	QUDB	Large cell lung carcinoma
**Breast cancer cell lines**		Calu6	Anaplastic carcinoma of lung
BT474	Invasive breast ductal carcinoma	Mehr	Pleural effusion
SKBR3	Pleural effusion from breast adenocarcinoma	**Kidney cancer cell line**	
MCF-7	Invasive breast ductal carcinoma	Hek-293	Human embryonic kidney
MDA-MB231	Pleural effusion from epithelial adenocarcinoma of breast	**Bladder cancer cell** **line**	
		Fen	Bladder epithelial cell
**Colorectal cancer cell lines**		**Prostate cancer cell lines**	
LS180	Caucasian colon adenocarcinoma	LNCaP	Prostate adenocarcinoma cells from lymph node metastasis
SW1116	Primary adenocarcinoma of Grade II extending to muscularis	**Ovarian cancer cell line**	
CACO2	Epithelial colorectal adenocarcinoma	Skov3	Metastatic ascitis- adenocarcinoma
SW742	Primary adenocarcinoma	**Head and neck cancer cell line**	
		HepII	Epidermoid carcinoma tissue from the larynx

## Materials and methods

**Cancer cell lines**

Table 1, lists the names and origins of 20 human cancer cell lines used in this study. The initial seed of all cancer cell lines were purchased from the National cell Bank of Iran, Pasteur Institute of Iran. Cancer cell lines from 10 different cancer types were cultured in RPMI1640 medium supplemented with 10% fetal bovine serum, 100 U/ml penicillin and 100 µg/ml streptomycin (Biosera, UK) in the standard culture condition. Cultures were maintained at <70% confluency by passage every 3-4 days. To perform the experiments, every culture was harvested and cells were divided into three sets. A fraction was sub-cultured in 24 well plates for analyzing the cellular growth characteristics. Another fraction was used for the extraction of total RNA and performing real time PCR for gene expression studies. The rest was used to extract cellular protein to measure the telomerase enzyme activity.

**Plotting growth curve and measuring doubling time of the cell lines**

Cell proliferation and doubling time was determined by seeding 1X10^4^ cells into wells of 24 well culture plates in quadruplicates. The amount of cell growth was measured by counting cells every 24 hours using Neobar counting chamber while excluding dead cells by 0.4% trypan blue vital dye. The doubling time of each cell line was calculated by plotting growth curve using online doubling time calculation freeware (http://www.doubling-time com/compute.php) (18).

**Table 2:** Sequence of primer pairs used for Real Time PCR evaluating hTERT variants and β-actin house keeping gene expression

**Position**	**Sequence**
Exo6 (F) primer	5'- TTG TCA AGG TGG ATG TGA CG -3'
Alpha (F) primer	5'- CTT TGT CAA GGA CAG GCT CA -3'
Exo7 (R) primer	5'- ATG TAC GGC TGG AGG TCT GT -3'
Beta (R) primer	5'- GGA CGT AGG ACG TGG CTC T -3'
β-actin (F)	5'- ACA GAG CCT CGC CTT TGC CG -3'
β-actin (R)	5'- CAC CAT CAC GCC CTG GTG CC -3'

**RNA isolation, cDNA synthesis and quantitative real time PCR**

**Fig. 1:**
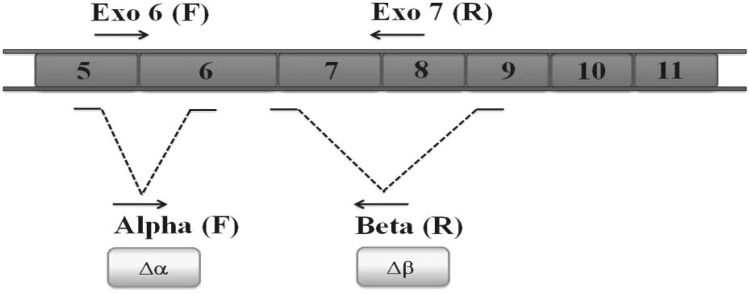
Deletion sites of different variants of hTERT Alpha deletion site is located on exon 6 while beta deletion site is on exons7 and 8. Arrows indicate the position of specific primers used to analyze the expression of full length hTERT and three alternatively spliced variants i.e. α-, ß- and αß- by real time PCR

Total RNA was extracted by TRIzol reagent (Invitrogen, USA) in accordance with the manufacturer’s instructions. The quality and quantity of the extracted RNA samples were determined by spectrophotometry. RNA samples were treated with DNase I (Fermentas, Lithuania) to remove genomic DNA contamination. cDNA was synthesized from 5 μg of the total RNA with the RevertAid H minus First Strand cDNA synthesis kit (Fermentas, Lithuania). The hTERT variant transcripts were measured through real time PCR using specific primers (Table 2) for each variant (α-deletion, β-deletion, α/β-deletion and full length variants) and SYBER Green I as reporter dye (Applied Biosystems, USA) on Chromo4 Detector thermal cycler (Bio-Rad, USA). The expression of β-actin was used as a housekeeping gene in all analyzes. Primer-Blast online freeware (http://www.ncbi.nlm.nih.gov/tools/primer-blast/) was used to design gene specific primers. Figure 1 shows the schematic location of hTERT gene deletions (∆αand ∆β) and also the binding sites of the designed primers on hTERT cDNA. Duplicated PCR reactions were set up in a final volume of 20 μL contained master mix, 0.5 µg of cDNA and 100 nM of each primer. The amplification program comprised an initial denaturation at 95 °C for 10 min and 45 cycles of denaturation at 95 °C for 15 s, annealing at 60 °C for 50 s, extension at 78 °C for 30 s. Fluorescence emission was collected at the end of the extension periods. The relative expression of hTERT variants was calculated by 2^-ΔCt^ equation. Melting curves were used to confirm the specificity of the fluorescent emission from the target sequences and rule out possible contamin-ation.

**Quantitative determination of telomerase activity**

To quantify telomerase activity in growing cells, the telomere length amplification protocol (TRAP) assay was performed using the TeloTAGGG Telomerase PCR ELISA kit (Roche, Germany) with three repeats, according to the manufacturers’ instructions. The kit contains biotin labeled synthetic primer and digoxigenin-(DIG) labeled detection probe specific for telomeric repeats. Briefly, telomerase available in the samples prepared from the cancer cell lines could add TAGGG repeats to the 3’-end of the biotin labeled primers during an amplification step. The PCR products were then denatured and hybridized to digoxigenin- (DIG) labeled detection probe. The resulting products were immobilized on streptavidin-coated microplate. Then, peroxidase conjugated antibody against DIG with tetramethy-lbenzisine (TMB) substrate were used for color formation in ELISA reaction. Presence of internal control ensured the accuracy of all reactions.

**Statistical analyzes**

The “one sample kolmogorov-Smirnov” test was used to determine the distribution of data. The Spearman’s correlation coefficient was used to find the relation between variables. P values less than 0.05 were considered as statistically significant in analyzes.

**Table 3:** The most abundant expressed variants of hTERT in the studied cancer cell lines

**Prevalent Variant**	**Cell line**
Full length	MCF-7, Mehr, HepII, Skov3, CACO2, MDA-MB231, QUDB, SKBR3, LNCap, BT474, Fen, SW1116, SW742, U87MG
α- variant	Hek293, LS180, Calu6, A172, KG-1
β- variant	Jurkat
α/β- variant	-

## Results

**Diverse doubling time among the studied cancer cell lines**

**Fig. 2:**
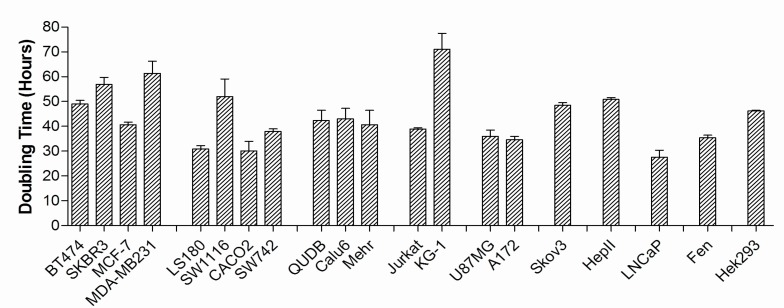
Doubling time of cancer cell lines Doubling time of different cancer cell lines were estimated by plotting the corresponding growth curves. The calculated time was used as index of proliferative capacity of the cell lines

Doubling times of the studied cell lines were measured by counting cells in the cultures and plotting growth curves. Figure 2 summarizes the doubling times of the 20 studied cell lines. As it is shown, KG-1 cells with myeloid origin had the longest doubling time (70 hours) which corresponds to slow proliferation kinetics of this cell line. On the other hand, LNCaP the prostate carcinoma cell showed the most rapid proliferation with doubling time of 28 hours followed by CACO2 and LS180 with doubling time of 30 h.

**Quantitative measurement of the hTERT varia-nts expression**

**Fig. 3:**
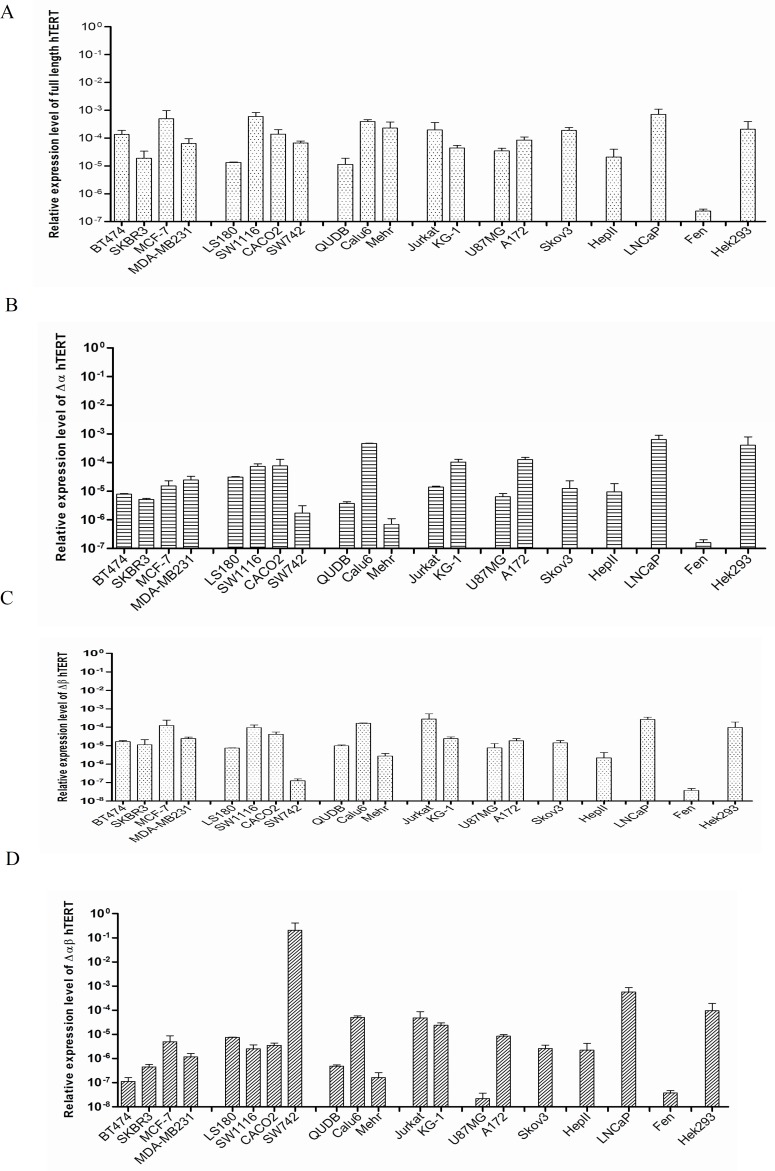
Relative expression levels of different hTERT transcript variants in the studied cancer cell lines**. **The expression of (A) full length, (B) α-deleted variant, (C) β-deleted variant and (D) α/β-deleted variants of hTERT were measured by real time PCR. The expression of the transcripts was compared to β-actin as internal housekeeping gene. All the studied cancer cell lines expressed a mixed pattern of all four hTERT variants

Quantitative real time PCR with specific primers for different variants of hTERT was set up to detect the expression levels of the hTERT variants in the studied cancer cell lines. Results showed high disparity in the relative proportions of hTERT transcript variants. Figures 3A, 3B, 3C and 3D represent the relative expression levels of the full length, α-deleted, β-deleted and α/β- deleted variants of hTERT in the cells, respectively. All the studied cancer cell lines expressed a combination of all four hTERT variants with no major restriction. When the expression of the variants were analyzed as percentages of the total hTERT messages in each cell lines, it showed that the full length variant in average encompass 56% of the transcripts (range from 22.70% to 98.46%) followed by α-deleted variant (mean 23%, range from 0.3%- 52%). The  β-deleted variant and α/β-deleted variant on average made up only 20% of hTERT expression in the studied cell lines. Table 3 lists the most prevalent hTERT variants expressed in the cell lines. As it is shown, the full length hTERT was the most predominant variant in the majority of the cells. Therefore, the ratios of expression level of this variant over the alternative spliced forms were measured to compare the hTERT composition of the cells. The median folds of excess expression of full length variant over the expression levels of α-deleted, β-deleted and α/β-deleted variants in the studied cells were 2.84, 3.7 and 31.7, respectively.

**Quantitative telomerase activity**

**Fig. 4:**
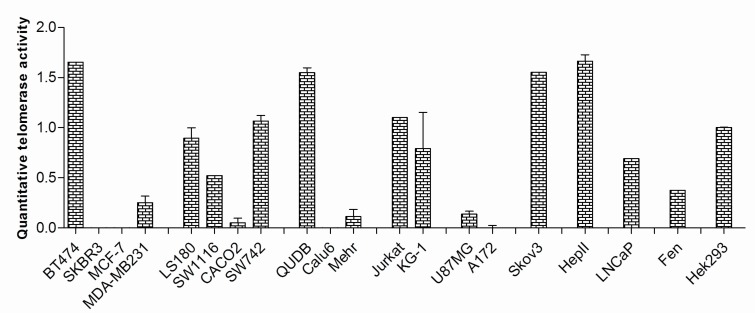
Telomerase enzyme activity in the cells was quantified by TRAP assay. The majority of the cells showed varying degrees of telomerase enzyme activity; however, four cell lines of different tissue origins failed to do that

TRAP assay was used to measure telomerase activity in the studied cell lines. Figure 4 demonstrates the corresponding quantities. As shown, significant telomerase activity could be detected in most cancer cells (80%); however, telomerase activity in four of the cancer cell lines, SKBR3, MCF-7, Calu6 and A172 could not be detected. To compare the expression levels of different variants in the cells with and without telomerase activity, the relative expression levels of full length transcript over the expression levels of other variants were determined. In the cells with detectable telomerase activity the mean folds increase in the expression of the full length variant over the α-deleted, β-deleted and α/β-deleted variants were 2.01, 3.10 and 4.40 respectively. The relative expression levels of full length transcript over the other variants in the cells without telomerase activity were 0.68, 4.61 and 10.17, respectively. The differences for α- deleted and α/β- deleted variants between the two cell groups were statistically significant (P<0.001 and P<0.005, respectively).

**Correlation between the expression of hTERT variants, the telomerase activity and prolifera-tion capacity**

**Fig. 5:**
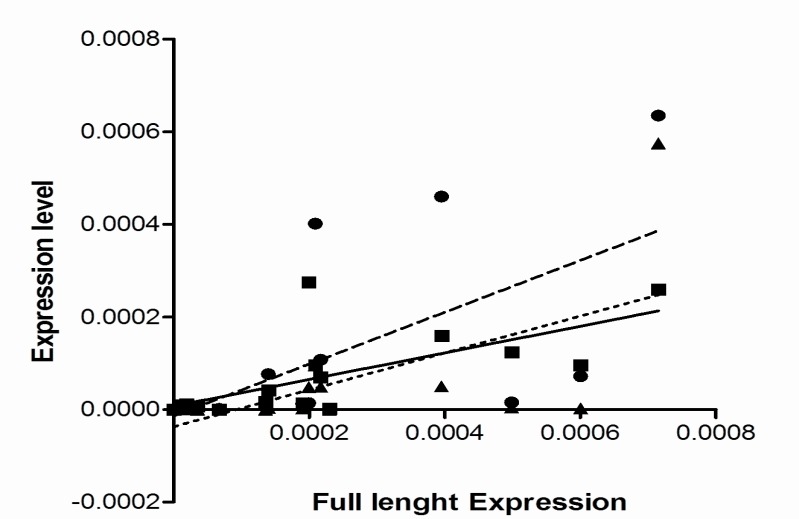
Correlation between the expression of full length hTERT variants and other alternative forms . The Spearman’s correlation coefficient was used to find statistical relation between the expression of full length hTERT transcript and the three spliced variants. The expression of full length transcript in the cells with telomerase activity was found in significant association with both β-variant (■ symbol, sold line, P= 0.0007, r= 0.74) and then α-variant (● symbol, broken line, P= 0.001, r= 0.7) but not α/β-deleted variant (▲ symbol, dotted line, P= 0.002, r= 0.68). There was no statistically significant correlation between the expression of the full length variant and other variants in the telomerase negative cells (P< 0.3, r< 0.3).

As it was shown in Figure 3 the full length hTERT variant was the most common variant expressed in the studied cell lines. Spearman’s correlation analysis showed that the expression of this variant in the cells with telomerase activity was in significant association with both β-variant (P= 0.0007, r = 0.74) and then α-variant (P= 0.001, r= 0.7). The expression of full length hTERT was in a weak correlation with α/β-deleted variant (P= 0.002, r= 0.68) (Figure 5). Similar study in the cells with no detectable telomerase activity could not show significant correlation between the expression of the full length variant (P<0.3, r<0.3). However, α-deleted variant had greater expression in telomerase negative cells (P= 0.04). No statistically meaningful correlation was found between telomerase activity and proliferation capacity (doubling time) of the cell lines (P=0.92, r= 0.0001). Similarly, no significant correlation between the expression of hTERT variants and telomerase activity or doubling time of the studied cancer cell lines was discovered.

## Discussion

Due to the importance of telomerase in cellu-lar multiplication process and its possible role in immortality of tumors, mechanisms regulating the enzyme activity have been of great attention for therapeutic purposes. In that regard, evidence dem-onstrated the importance of the alternative splicing forms of hTERT transcripts in the regulation of telomerase activity (19) although controversies exist (4). In our study of 20 established cancer cell lines, there was a wide variation (28 h to 70 h) among doubling times of the cells. Such variation was in line with other reports in the literature. The BioGPS database shows the great variation of 19 h to 67 h for the doubling time of 174 cancer cells taken from 9 different human tissues (20).

Among cell lines that we studied, 80% showed detectable telomerase activity while MCF-7, SKBR3, Calu6 and A172 failed to do so. Although most tumor cells retain telomerase activity, some others may use alternative mechanisms such as recombination mediated DNA replication to lengthen their telomeres and prevent chromosomal erosions during cell division (21). Such mechanisms have been shown to be more common in cancer cells with mesenchymal origin but not in cells with epithelial origin (22). In our study, however, the telomerase negative cell lines were originated from different embryonic germinal layers (MCF-7 and SKBR3, ectodermal; Calu6, endoderm). Many investigations have tried to show correlation between proliferation capacity and telomerase enzyme activity in tumor cells (23). In agreement with Hoos et al. our findings however, did not show such correlation (24). In fact, despite the expression of telomerase in most tumors, significant telomere shortening in cancer cells has been reported (25-26). Different kinetics of telomerase activity in cells with shorter or longer telomere lengths have been shown (27). Literature shows diverse results regarding the expression pattern of hTERT in different cancer cells (28-29). When we assessed the patterns in the studied cell lines, the full length hTERT spliced variant was found as the dominant type. Similar findings have been reported in other studied tumor cells of different origin such as breast cancer, leukemias and myelodysplastic syndromes (16, 29-31). Despite that, there are other reports showing the preferential over-expression of other variants in different cancer cells. In this regard, β alternative splice variant was found to be more expressed in some gastric cancers (32). Due to the regulatory role of hTERT variants on telomerase activity, many reports showed correlation (direct or reverse) between telomerase activity and the expression of some hTERT variants in tumor cells (12, 29, 33). In our study however, there was no correlation between the expression of alternative splicing variants, telomerase enzyme activity and proliferative capacity of the cells from any histological or embryological origin. The ratio of the expression level of the full-length hTERT transcript over the expression levels of other alternative spliced variants was used to predict telomerase activity in cancer cells like lung and melanomas (34). When we compared the ratios among the cells with and without telomerase activity, it showed that α-deleted transcript comprised higher proportions of hTERT transcripts in the telomerase negative cells. On the other hand, in these cells α/β-deleted variant were much limited in comparison to other splice transcripts. This may point toward the inhibitory role of α-deleted variants in the cells to suppress telomerase activity. Listerman et al. also demonstrated that β-deleted variant may compete for binding to telomerase RNA, thereby inhibiting the overall endogenous telomerase activity (16). However, more results are needed to support these findings.

All together, data show that cancer cells do not share a unique mechanism for the expression of the hTERT variants to regulate telomerase activity. It has to be mentioned that the tested cells in this study were established cell lines which could affect the pattern of telomerase activity and hTERT expression. Despite the complexity of hTERT and telomerase machinery, they still have great potentials to be exploited as targets for cancer therapies. Therefore, more cells from same tissue origins or cancer tissues should be tested to draw a comprehensive conclusion.
